# Effect of sodium lactate on the gel quality of cold-stored *Litopenaeus vannamei*: Perspective of 4D-DIA proteomics

**DOI:** 10.1016/j.fochx.2026.103835

**Published:** 2026-04-05

**Authors:** Yantao Yin, Huixiang Ye, Jia Cai, Caixia Yin, Tengyang Guo, Naiyong Xiao, Zefu Wang, Shuai Wei, Shucheng Liu

**Affiliations:** a College of Food Science and Technology, Guangdong Ocean University, Guangdong Provincial Key Laboratory of Aquatic Product Processing and Safety, Guangdong Provincial Engineering Laboratory for Marine Biological Products, Guangdong Provincial Engineering Technology Research Center of Seafood, Zhanjiang 524088, China; b Collaborative Innovation Center of Seafood Deep Processing, Dalian Polytechnic University, Dalian 116034, PR China

**Keywords:** White shrimp, Shrimp surimi gels, Sodium lactate, Proteomics

## Abstract

The aim of this study was to investigate the effect of sodium lactate (SL) on the gel quality of cold-stored *L.vannamei* and its underlying mechanism. Fresh *L.vannamei* were soaked in 1%, 2%, 3% SL solution (or pure water as the control, HO group) for 30 min, followed by 3 days' cold storage at 4 °C. The results showed that cold storage significantly deteriorated the gel quality, compared with the fresh shrimp (FS group), and the HO group exhibited a significant decrease in gel strength and an increase in cooking loss (*p* < 0.05). In contrast, SL treatment alleviated these quality defects in a concentration-dependent manner, with the 3% SL group showing the most prominent improvement. Proteomic analysis identified 1572 proteins in total. Compared with the FS group, the HO group had 174 differentially abundant proteins (DAPs), mainly involving degradation of structural proteins (e.g., myosin heavy chain, profilin, tubulin beta-2 chain), downregulation of energy metabolism enzymes (e.g., pyruvate kinase, arginine kinase), reduction of antioxidant proteins (e.g., glutathione peroxidase, copper/zinc superoxide dismutase), and impairment of immune-related proteins (e.g., prophenoloxidase-1, C-type lectin 2, penaeidin-3a). When compared with the HO group, the 3% SL group had 83 DAPs (65 upregulated, 18 downregulated), including significant upregulation of the aforementioned structural, energy metabolism, antioxidant, and immune-related proteins. The measurements of thiobarbituric acid reactive substances and protein carbonyl confirmed that SL significantly reduced oxidative damage in the 3% SL group compared with the HO group. Take together, SL attenuated protein degradation, protected energy metabolism homeostasis, and alleviated oxidative stress, thus improving the gel quality of cold-stored *L.vannamei*.

## Highlights

• Cold storage impaired gel quality of L. *vannamei.*
• Sodium lactate improved the gel quality of cold-stored *L. vannamei.*
• Sodium lactate reduced structural proteins degradation of cold-stored *L. vannamei.*
• Sodium lactate maintained energy and immune homeostasis of L. *vannamei* during cold storage.

## Graphical abstract

**Figure:**
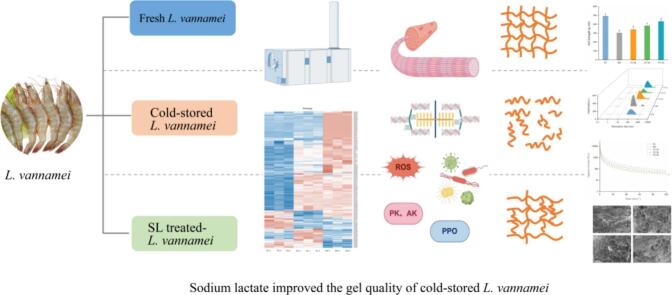

## 1 Introduction

*Litopenaeus vannamei* (*L. vannamei*), as one of the world's highest yielding economic crustacean aquatic products, plays a crucial role in aquaculture and food processing industries. According to the Food and Agriculture Organization of the United Nations (FAO), the global production of L. *vannamei* was 6.8 million tonnes in 2022 (FAO, 2024). In the field of food processing, *L. vannamei* is widely used in the production of gel processed products such as shrimp balls, shrimp cakes, shrimp slides, etc. (Pan et al., 2024). Such kind of products are favored in the consumer market because of their soft taste and convenient eating.

However, the gel quality of *L.* *vannamei* during fishing, transportation and cold storage is vulnerable to the influence of microbial activities and endogenous enzymes, resulting in excessive protein hydrolysis, which severely restricts its processing applicability and product quality (Peng et al., 2022). Specifically, under cold storage, although the growth rate of microorganisms is inhibited, low-temperature spoilage bacteria such as *Pseudomonas* and *Shewanella* can still grow and secrete proteases to degrade myofibrillar proteins in shrimp meat (He et al., 2024). At the same time, the endogenous enzymes (such as cathepsin B, L, D) contained in *L.* *vannamei* still have high activity under cold storage, which can degrade myosin, reduce its ability to form a three-dimensional gel network during heating, and thus reduce the taste of the shrimp gel products (Peng et al., 2019). On the other hand, the destruction of protein structure will lead to the decrease of its water holding capacity (Wu et al., 2024). The product is prone to water loss during processing and storage, which not only leads to a decline in product yield, but also makes the gel structure loose and tastes rough (Huang, Wang & Tu, 2023). Therefore, how to effectively inhibit the activities of spoilage microorganism and endogenous proteolytic enzymes during the cold storage of *L.* *vannamei*, and thus improve its gel quality needs to be urgently solved.

Sodium lactate (SL) has been designated as generally recognized as safe (GRAS) for use as a key emulsifier, flavor enhancer, humectant, and pH regulator under established good manufacturing practices (GMPs), as specified in CFR 184.1768 (Cho & Kang, 2022; Yang et al., 2025). Initially, SL was mainly used for the preservation and quality improvement of meat products such as sausages and ham. It can be used to reduce the water activity (Aw) of food, adjust the pH value, inhibit microbial growth, and combine with proteins to enhance the water holding capacity of proteins, thereby improving the tenderness and juiciness of products (Cho & Kang, 2022). In recent years, the application of SL in the aquaculture, processing, and preservation of aquatic products has gradually expanded. Li et al. (2022) reported that SL supplementation in Nile tilapia diets enhanced protein and lipid synthesis. Yang et al. (2025) reported that the application of 5% SL in the salting process of *Larimichthys crocea* markedly enhanced the water-holding capacity and sensory acceptability. However, there is few studies about the effect of SL on *L.* *vannamei*, especially in the gel quality of *L.* *vannamei* during cold storage.

4D-DIA proteomics is an advanced proteomic technique that integrates ion mobility as a fourth dimension alongside traditional mass spectrometry dimensions. It enables comprehensive analysis of protein expression and composition in biological samples while delivering exceptional resolution and quantitative accuracy, which allows researchers to capture the full molecular diversity of complex proteomes with unprecedented depth and reliability (Liu et al., 2026).

Based on the above research background, this study will investigate the effect of SL on the gel quality of cold-stored *L.* *vannamei* from the perspectives of gel strength, cooking loss, water distribution, apparent viscosity, and microstructure. In addition, 4D proteomics was adopted to analyze the potential mechanism of SL affecting the gel quality of cold-stored *L. vannamei*. The findings will help enhance the gel quality of *L.* *vannamei* and facilitate the high-value development of the shrimp processing industry.

## 2 Materials and methods

### 2.1 Sample preparation

Fresh *L.* *vannamei*, with a specification of 30–40 tails/kg, purchased from Xiashan Water Products Wholesale Market in Zhanjiang, Guangdong Province, China. The shrimps were immediately transported to the laboratory under oxygen-supplemented conditions. After removing the head, shell, and intestinal glands, the shrimp were soaked in 1%, 2%, and 3% concentrations of SL (referred to as 1% SL, 2% SL, and 3% SL group, respectively) for 30 min. The soaking ratio was 1:3 (w: w), and the soaking temperature was 4 °C. At the same time, pure water soaking (referred to as the HO group) was used as a control. After soaking, store each group of shrimps in a 4 °C freezer for 3 days, and then make shrimp surimi.

### 2.2 Preparation of shrimp surimi

Fresh *L.* *vannamei* (designated as the FS group) was used in this study, together with HO, 1% SL, 2% SL, and 3% SL samples that had been subjected to 3 days of cold storage. Shrimp surimi was prepared following our previously method (Liang, Jiang, et al., 2025). Briefly, the shrimp meat was rinsed three times with ice-cold water at a volume five times that of the meat, rinsing for 3 times, each time for 10 min. The shrimp meat was dehydrated with cotton gauze, and then the shrimp meat was thoroughly minced using a blender (MQ785, Braun, Germany) at 12,000 rpm for 3 min. Subsequently, 3% NaCl was incorporated into the minced meat, which was then minced at 12,000 rpm for another 3 min. The resulting shrimp mince was further processed until a moisture content of 78% was achieved. Briefly, the initial moisture content of each minced sample was determined according to the ISO 1442:2023. Based on the initial reading, an appropriate amount of ice-cold 3% NaCl solution was added to achieve a final moisture content of 78% (*w*/w). The mixture was then homogenized at 4000 rpm for 3 min to ensure uniform distribution.

### 2.3 Preparation of shrimp surimi gel

The preparation of heat-induced shrimp surimi gel was performed as described by Liang, Jiang, et al. (2025), with slight modifications. Briefly, 30 g of shrimp surimi was packed into 50 mL centrifuge tubes and heated in a water bath via a two-step heating protocol: first at 40 °C for 30 min, followed by heating at 90 °C for another 30 min. Subsequent to heating, the shrimp surimi gels were rapidly cooled using an ice-water mixture.

### 2.4 Gel strength

A TMS-Pro texture analyzer (FTC, Virginia, USA) was used to determine the gel strength according to the method described by Cao et al. (2020). The test parameters were set as follows: probe type: P/0.5 s; trigger force: 5.0 g; test speed: 1 mm/s; pre-test and post-test speeds: 2 mm/s.

### 2.5 Cooking loss

Cooking loss was calculated via the following Eq. (1):

(1) $$\text{Cooking loss}\;\left(\%\right)=\left(M_{1}-M_{2}\right)/M_{1}\times 100\%$$

M_1_ denotes the weight of shrimp surimi prior to heating, while M_2_ corresponds to the weight of shrimp surimi gel following two-stage heating.

### 2.6 LF-NMR

In line with the method described by Liang, Jiang, et al. (2025), the water distribution of shrimp surimi gel was measured using a low-field nuclear magnetic resonance (LF-NMR) spectrometer (NMI21-060H, Newman, Suzhou, China). First, the shrimp surimi gel was cut into columnar specimens with a height of 10 mm and a diameter of 20 mm. These specimens were then placed into an NMR tube. Measurements were performed using the CPMG (Carr-Purcell-Meiboom-Gill) pulse sequence. Parameters were set as follows: the repetition interval was configured to 4000 ms, and the number of echoes was set to 3000. Subsequently, the relaxation time ranging from 0.01 to 3000 ms was fitted. Lastly, the data were inverted using MultiExpInv analysis software.

### 2.7 Rheology

The rheological properties of shrimp surimi were measured using a rheometer (Haake Mars 60, Thermo Fisher, Germany) in accordance with the protocol described by Liu et al. (2022). Shear experiments were performed at 25 °C using parallel plates (35 mm diameter, 1 mm gap). Shear rates were set between 0.1 and 100 s^−1^ for the quantification of apparent viscosity.

### 2.8 Sem

According to the protocol described by Cao et al., 2020, the shrimp surimi gel was first cut into small cubic pieces measuring 3 mm × 3 mm × 5 mm. Subsequently, these pieces were immersed in 0.1 mol/L phosphate buffer (pH 7.2) supplemented with 2.5% (*v*/v) glutaraldehyde and incubated for 24 h. After this incubation step, the samples were rinsed three times with 0.1 mol/L phosphate buffer (pH 7.2) prior to undergoing a gradient dehydration process using a sequence of ethanol solutions. Scanning electron microscopy (SEM; MIRA LMS, Tescan, Brno, Czech Republic) was used to acquire microstructural images of the shrimp surimi gel. The accelerating voltage was adjusted to 2 kV, while the magnification was set to 3000 × .

### 2.9 Proteomic analysis

#### 2.9.1 Total protein extraction

Proteomic analysis was conducted on samples of FS, HO, and 3% SL. Protein sample was extracted from shrimp abdominal muscle. Then, protein samples were cryogenically pulverized in liquid nitrogen, followed by homogenization in lysis buffer containing 8 M urea, 0.1% SDS, and 1% protease inhibitor cocktail using a high-throughput tissue grinder (3 cycles × 180 s each). Subsequent non-contact ultrasonic disruption (30 min) was performed at cryogenic temperatures. The resulting lysate underwent centrifugation at 16,000 *g* for 10 min at 4 °C, with the supernatant retained for downstream analysis.

#### 2.9.2 Protein digestion

A total of 100 μg protein was dissolved in 100 mM triethylammonium bicarbonate (TEAB) buffer. The solution underwent reduction by adding 10 mM Tris (2-carboxyethyl) phosphine (TCEP) followed by incubation at 37 °C for 60 min. Alkylation was then performed using 40 mM iodoacetamide (IAM) under dark conditions at room temperature for 40 min. After centrifugation at 10,000 ×*g* for 20 min at 4 °C, the resulting pellet was reconstituted in 100 μL of 100 mM TEAB buffer. Trypsin digestion was initiated at an enzyme-to-substrate ratio of 1:50 (*w*/w) with overnight incubation at 37 °C. Following this, the peptide mixture was lyophilized and desalted prior to further analysis.

#### 2.9.3 DIA mass detection

The peptide quantification was performed using a Vanquish Neo ultra-high-performance liquid chromatography (UHPLC) system interfaced with an Orbitrap Astral mass spectrometer (Thermo Fisher Scientific, USA). Separation was achieved using a uPAC High Throughptu analytical column (75 μm × 5.5 cm, Thermo Fisher Scientific) with mobile phases consisting of solvent A (2% acetonitrile/0.1% formic acid in water) and solvent B (80% acetonitrile/0.1% formic acid in water), employing an 8-min gradient elution program. Mass spectrometric analysis was conducted in data-independent acquisition (DIA) mode with a mass-to-charge ratio (*m*/*z*) scanning range of 100–1700.

#### 2.9.4 Protein identification and bioinformatic analysis

The DIA raw data were analyzed using Spectronaut software (Version 19) based on the database (https://www.uniprot.org/taxonomy/6689). With the following parameters: peptide length range (7–52 amino acids), trypsin/P as the cleavage enzyme (max 2 missed cleavages), fixed carbamidomethylation of cysteines, and variable modifications including methionine oxidation and protein N-terminal acetylation. Protein identification thresholds were set at FDR ≤0.01, with peptide-level requirements of FDR ≤0.01, confidence ≥99%, and XIC width ≤ 75 ppm. Protein quantification was performed using the Max LFQ algorithm. The bioinformatic analysis of the proteomic dataset was conducted via the Majorbio Cloud platform. For proteins across the two groups, *p*-values and Fold Change (FC) were computed utilizing the R package “*t*-test”. Differentially abundant proteins (DAPs) were identified by applying the thresholds of FC (>1.2 or < 0.83) and p-value <0.05.

### 2.10 Statistical analysis

All experiments were performed in triplicate, and the results were expressed as mean values with the associated standard deviations. For statistical analysis, Duncan's multiple range test was employed with SPSS software (version 22.0, SPSS Inc., Chicago, IL, USA). Statistical significance was defined as a p-value <0.05.

## 3 Results and discussion

### 3.1 Changes in gel strength

**Fig. 1:**
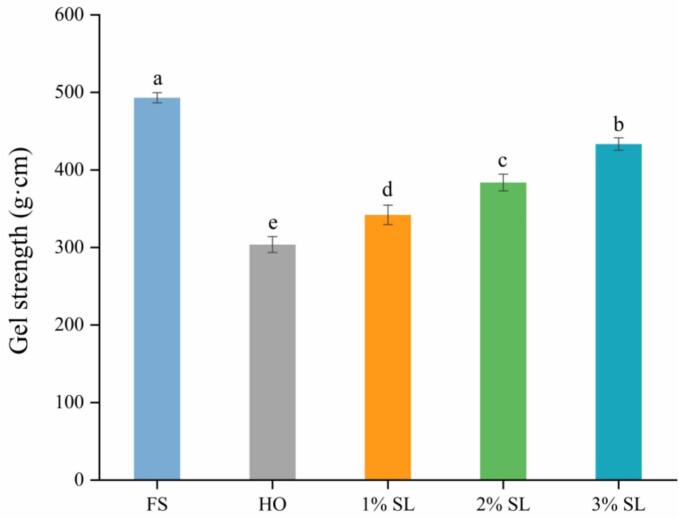
Effect of SL on the gel strength of L. *vannamei* in different groups. Note: the symbol (a, b, c, d, e) denotes statistically significant differences between the two groups.

The gel strength gel is and an important indicator to determine the quality of shrimp surimi products such as shrimp balls, and shrimp cakes. As shown in Fig. 1, compared with FS, cold storage significantly reduced the gel strength of *L.* *vannamei*. Specifically, compared with FS group, the gel strength of the HO group decreased by 38.41%. Similarly, Liang, Sun, et al. (2025) reported that cold storage reduced the texture quality of *L.* *vannamei*. This phenomenon might be due to protein hydrolysis and oxidation during cold storage, thereby reducing its gel strength (Zhu et al., 2024). Meanwhile, SL group effectively improved the gel strength of cold-stored L. *vannamei*, and with the increase of SL concentration, its gel strength also gradually increased (Fig. 1). Specifically, the gel strength of the 3% SL group increased by 42.69% compared with HO group, suggesting that SL could effectively inhibit the deterioration of *L.* *vannamei* during cold storage. The protective effect of SL on the quality of *L.* *vannamei* gel might be related to its inhibition of the growth of spoilage bacteria. Schelegueda et al. (2016) reported that SL effectively inhibited *S. putrefaciens* and psychotropic bacteria in fish meat. In addition, lactate ion, as kosmotropic ion, has strong hydration ability, which could promote the formation of a more stable interaction network between polar groups of protein molecules and water molecules (Min et al., 2023). Simultaneously, Na^+^ released from sodium lactate increases ionic strength, facilitating myosin tail unfolding and exposing reactive sites for cross-linking. Furthermore, lactate ion could combine with charged groups on the surface of protein, stabilizing protein structure (Yang et al., 2025). These physicochemical effects likely act in concert with the biological mechanisms to improve gel strength of cold-stored *L.* *vannamei*.

### 3.2 Changes in cooking loss and water distribution

**Fig. 2:**
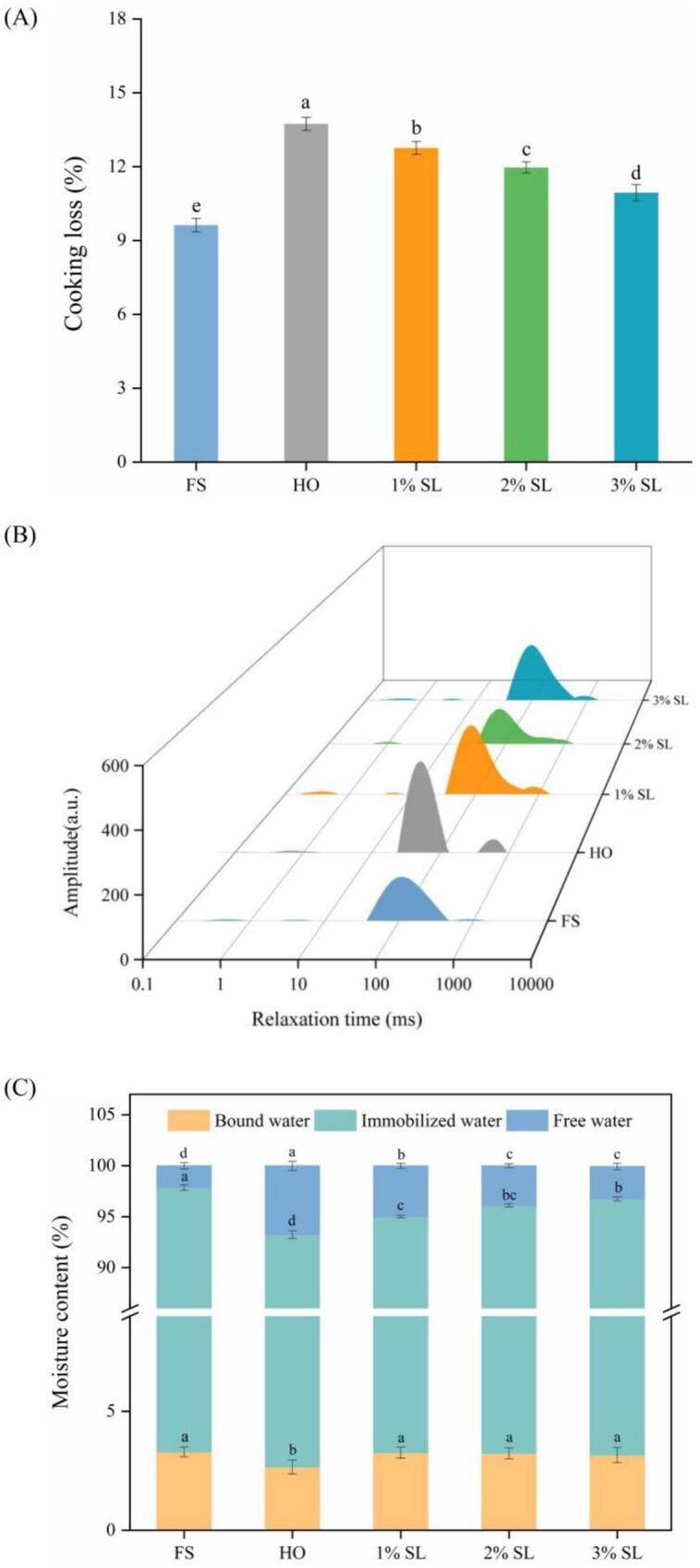
Effect of SL on the gel cooking loss and water distribution in different groups. Note: the symbol (a, b, c, d, e) denotes statistically significant differences between the two groups.

The water binding capacity of shrimp surimi gel determines the juiciness, tenderness and flavor retention. And it also directly affects the product yield and cost control of manufacturers. As shown in Fig. 2A, cold storage significantly increased the cooking loss of *L.* *vannamei* gel. Compared with FS, the cooking loss of HO group increased by 42.73%. As expected, SL remarkably alleviated the increase of cooking loss caused by cold storage (*p* < 0.05). The cooking loss of 3% SL group decreased by 20.33%, compared with HO group. LF-NMR has emerged as a widely employed analytical technique for investigating the water distribution characteristics and migration behaviors of water fractions with distinct binding states in food systems (Liang, Jiang, et al., 2025). As shown in Fig. 2B, three peaks were detected in each group of samples, representing bound water, immobile water, and free water from left to right. As shown in Fig. 2C, the proportions of bound water, immobile water, and free water in FS sample was 3.29%, 94.57%, 2.14%, respectively. Compared with FS group, the proportion of bound water, immobilized water in HO group were significantly decreased, while the proportion of free water significantly increased (*p* < 0.05). Compared with the HO group, more bound water, and immobilized water, while less free water were observed in the SL groups, especially in the 3% SL group (p < 0.05).

The increase of cooking loss in HO group might be due to the degradation of proteins during cold storage, which reduced their ability to bind water (Wu et al., 2024). In addition, the degradation and denaturation of protein during cold storage led to the deterioration of gel network structure, which made it difficult to lock water, thus increasing the cooking loss (Zhu et al., 2024). We speculate SL reduced the degree of protein hydrolysis in shrimp muscle during cold storage, consequently mitigating cooking loss (Fig. 5). In addition, the strong hydration capacity of SL facilitated the formation of a dense hydration layer on the surface of protein molecules, which in turn enhanced the water retention capacity of shrimp muscle and thereby contributed to the reduction of cooking loss (Yang et al., 2025).

### 3.3 Changes in apparent viscosity

**Fig. 3:**
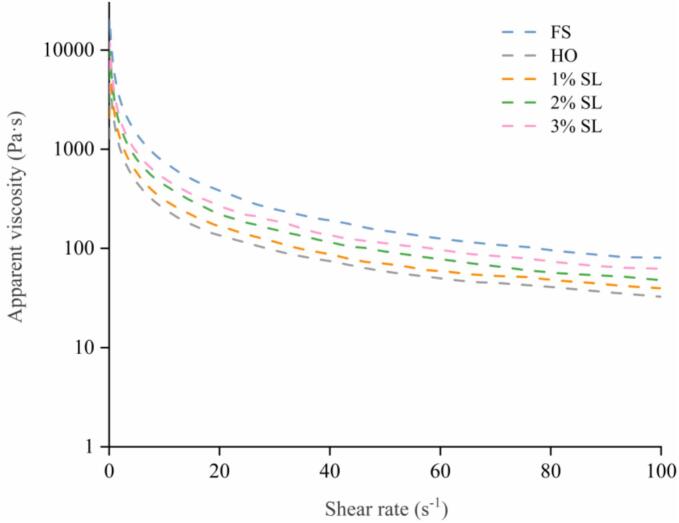
Effect of SL on the apparent viscosity of shrimp surimi in different groups.

The apparent viscosity can reflect the strength of intermolecular interactions between myofibrillar proteins in shrimp surimi system and the potential for three-dimensional network construction. As shown in Fig. 3, compared with the FS group, the apparent viscosity of HO group significantly decreased (*p* < 0.05). The decreased apparent viscosity might be related to the degradation of shrimp meat protein during cold storage, which resulted in lower intermolecular friction and network strength of shrimp surimi. Similarly, Peng et al. (2019) reported that the apparent viscosity of shrimp gradually decreases during refrigeration. Compared with the HO group, SL treatment significantly increased the apparent viscosity of shrimp surimi, and the apparent viscosity increased with the increase of SL concentration. The increase in apparent viscosity could be attributed to SL protecting the integrity of myofibrillar protein. In addition, the Na^+^ released by SL could increases the ionic strength of the shrimp surimi system, which in turn facilitated the unfolding of myosin tails, thereby exposing more active sites and ultimately strengthening intermolecular cross-linking (Li et al., 2024).

### 3.4 Changes in microstructure

**Fig. 4:**
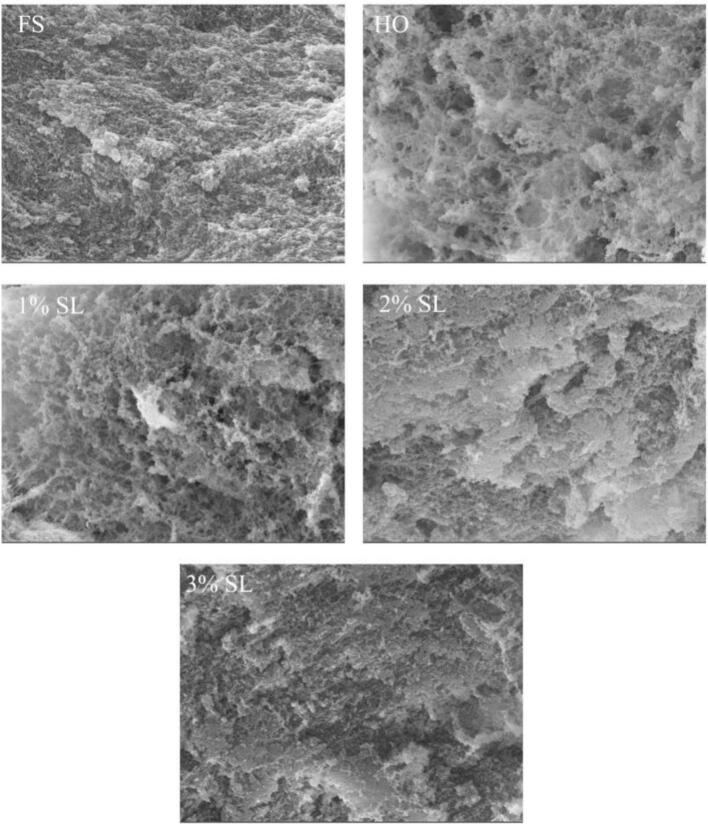
Effect of SL on the gel microstructure of L. *vannamei* in different groups.

The quality of shrimp surimi gel is closely related to its microstructure. As shown in Fig. 4, the microstructure of FS group was evenly and densely arranged, and the surface is flat, suggesting that fresh shrimp could form a good gel network. Compared with the FS group, the microstructure of the HO group was loose and disordered, with numerous pores of varying sizes. This was mainly related to the degradation and denaturation of myosin during cold storage, which reduces the ability to form network gel (Zhang et al., 2023). Compared with the HO group, the pores in the 1% SL group were reduced and the structure was relatively compact, and the microstructure of 2% SL group was further improved. Compared with the 1% SL and 2% SL groups, the microstructure of the 3% SL group continued to develop towards a dense and orderly direction, with a more regular network structure and tight and uniform connections between protein molecules. These changes in microstructure further indicated that SL could improve the gel quality of cold-stored *L.* *vannamei*.

### 3.5 Proteomics

**Fig. 5:**
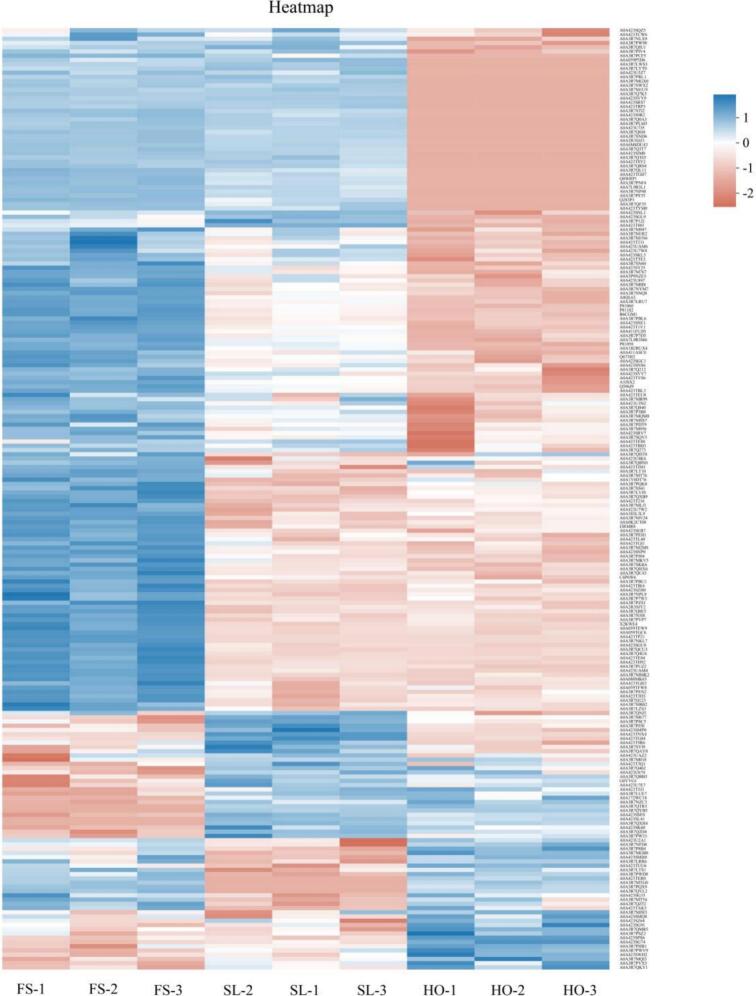
Heatmap of identified proteins in the FS, HO, and 3% SL group.

According to the above results of gel strength, cooking loss, apparent viscosity and microstructure, 3% SL had the best effect on improving the gel quality of cold-stored *L. vannamei*. Therefore, the FS, HO, and 3% SL groups were selected for the proteomic analysis. As shown in Fig. 5, a total of 1572 proteins were identified in this study, which was much higher than that reported by Zhang et al. (2020). This might be due to the method used in this study was 4D-DIA proteomics, which could provide deeper sequencing. The full list of 1572 identified proteins with their expression values across all groups is provided in Supplementary 1.

**Fig. 6:**
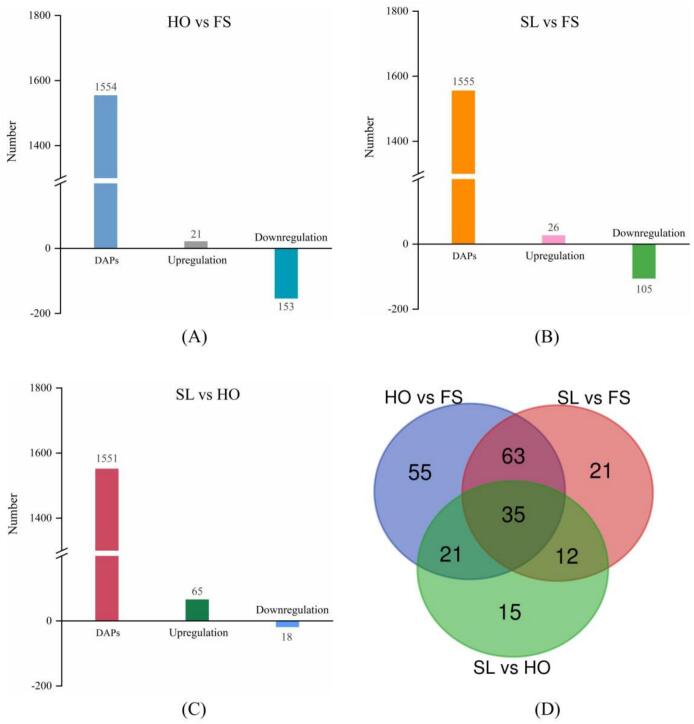
DAPs in different groups. (A): HO vs FS, (B): SL vs FS, (C): SL vs HO, (D): Venn diagram of different groups.

Among the 1572 identified proteins, a total of 174 DAPs were identified in the HO vs FS groups (Fig. 6A). Specifically, compared with the FS group, 21 proteins were upregulated and 153 proteins were downregulated in the HO group. These large amounts of downregulated proteins suggested that the protein in *L.* *vannamei* underwent severe degradation during cold storage (Wu et al., 2024). Contrastively, as for 3% SL vs FS groups, 131 DAPs were identified; 26 proteins were upregulated, and 105 proteins were downregulated (Fig. 6B). At the same time, compared with the HO group, 65 proteins were upregulated and 18 proteins were downregulated in the 3% SL group (Fig. 6C). Especially, as shown in the Venn diagram (Fig. 6D), there were 56 same of DAPs between the HO vs FS, and 3% SL vs HO groups. These results suggest that, compared with the HO group, the proteome of 3% SL sample was much closer to FS sample, and this could also be seen from the heatmap (Fig. 5).

**Fig. 7:**
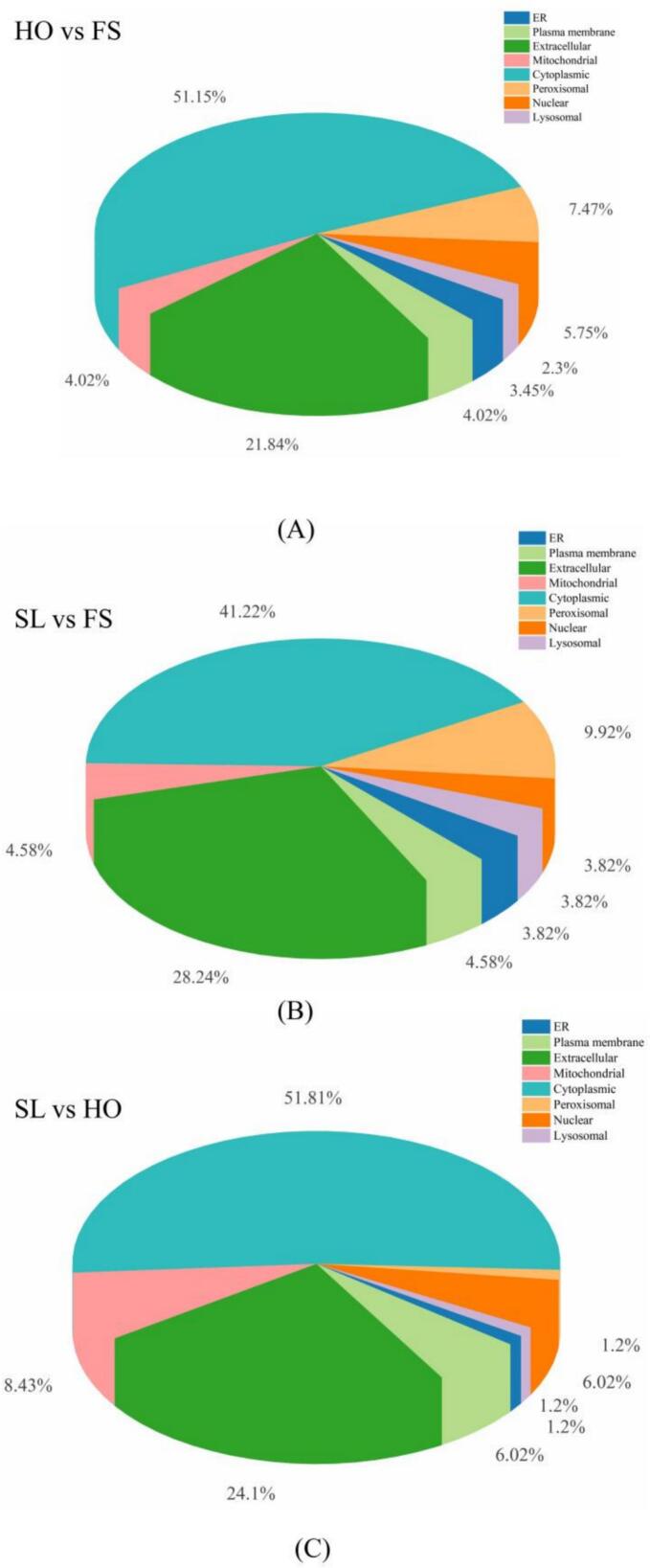
Classification of the DAPs according to their localization. (A): HO vs FS, (B): SL vs FS, (C): SL vs HO.

To understand the functionality of these DAPs, subcellular localization was performed, and the results are shown in Fig. 7. It was noted that the DAPs were mainly distributed in the cytoplasmic and extracellular regions. Specifically, in the HO vs FS group, 51.15% of DAPs were located in the cytoplasmic region, and 21.84% of DAPs were located in the extracellular region (Fig. 7A). In the SL vs FS group, 41.22% of DAPs were located in the cytoplasmic region, and 28.24% of DAPs were located in the cytoplasmic region (Fig. 7B). In the SL vs HO group, 51.81% of DAPs were located in the cytoplasmic domain, and 24.10% of DAPs were located in the extracellular domain (Fig. 7C). The cytoplasmic proteins mainly involve skeletal proteins, such as myosin heavy chain, actin (Yu et al., 2023). The extracellular proteins mainly involve immune related proteins, such as Penaeidin, Alpha-2-macroglobulin (Sun et al., 2023). Therefore, it is reasonable to infer that the skeletal proteins and immune proteins might play a dominant role in the quality deterioration of *L.* *vannamei* during cold storage, and SL could effectively inhibit the degradation of such proteins.

In order to further explore how these DAPs improved the gel quality of chilled *L.* *vannamei*, we classified these DAPs according to their possible functions (Table 1), and corresponding discussions were conducted as bellow.

**Table 1:** DAPs of L. *vannamei* in HO vs FS, and SL vs HO group.

Accession	Protein Name	Fold change	
		HO vs FS	SL vs HO
Structural proteins			
A0A423T881	Myosin heavy chain type 1	down	up
A0A3R7MR99	Myosin heavy chain type 2	down	no
A0A3R7SNQ8	Myosin heavy chain type a	down	no
A0A3R7P1J2	Putative myosin heavy chain, muscle-like	down	up
A0A423U897	Putative filamin-A isoform X3	down	no
A0A423SRV7	Arp2/3 complex 34 kDa subunit	down	no
A0A1B2RUX4	Profilin	down	up
A0A3R7MGX0	Tubulin beta-2 chain	down	up
A0A3R7PJ50	Myosin heavy chain type 2	no	up
A0A423TG84	Myosin heavy chain type 1	no	up
A0A3R7PSC5	Myosin	no	up
A0A3R7QXH4	Myosin heavy chain type 2	no	up
A0A423SQZ5	Muscle M-line assembly protein unc-89	no	up
Energy metabolism related proteins			
A0A3R7LWS3	Arginine kinase	down	up
A0A423T8Y2	Carnitine O-palmitoyltransferase	down	up
A0A5P9NZE5	Phosphoglycerate kinase	down	no
A0A3R7QR84	Propionyl-CoA carboxylase alpha chain, mitochondrial	down	up
A0A423U7J5	Pyruvate kinase	down	up
Oxidative stress related proteins			
A0A3R7LVJ8	Glutathione peroxidase	down	no
A0A3R7PJ04	Glutathione peroxidase 6	down	no
A0A3R7PW98	Glutathione S-transferase 1	down	up
A0A423TH92	Copper/zinc superoxide dismutase isoform 4	down	no
A0A411ASC0	Hypoxia up-regulated protein	down	no
A0A059P5D6	Metallothionein	no	up
A0A3R7Q0J4	Peroxinectin	no	up
A0A3R7M1U9	Elongation factor 1-alpha	no	up
proPO system related proteins			
A0A3R7LRU7	Prophenoloxidase-1	down	up
A5JSX2	Prophenoloxidase 2	down	up
A0A3R7PF55	Prophenoloxidase activating factor	down	up
A0A423S9R2	Alpha-2-macroglobulin	down	up
A0A3R7QC43	Hemocytin	down	no
A0A3R7P7D5	Putative hemocytin	down	up
A0A3R7QL11	JHE-like carboxylesterase 1	down	up
Immune recognition related proteins			
A0A3R7PQX9	C-type lectin 2	down	no
A0A423TY06	C type lectin containing domain protein	down	up
A0A3R7PLM5	Beta-1,3-glucan binding protein	down	up
A0A2R3SJZ1	Anti-lipopolisaccharide factor 5	down	up
Q283P3	Anti-lipopolysaccharide VV-R isoform	down	up
Antimicrobial peptide related protein			
P81058	Penaeidin-3a	down	up
P81060	Penaeidin-3c	down	up
Q596J9	Crustin I	down	up
A0A3R7SND6	Crustin-like protein	down	up
A0A6M4DU43	BigPEN	down	up
Q8WRP1	Putative antimicrobial peptide	down	up

#### 3.5.1 Structure-related DAPs

The structural integrity of myofibrillar protein has a decisive influence on the quality of shrimp surimi gel (Zhang et al., 2023). In this study, compared to the FS group, myosin heavy chain type 1, myosin heavy chain type 2, myosin heavy chain type a, putative myosin heavy chain, muscle-like, putative filamin-A isoform X3, Arp2/3 complex 34 kDa subunit, profilin, tubulin beta-2 chain were significant decline in the HO group.

Myosin heavy chain type 1, myosin heavy chain type 2, myosin heavy chain type a, and putative myosin heavy chain, muscle-like belong to different subtypes or homologous proteins of the myosin family (Wang et al., 2023). As is known to us, myosin plays a key role in the formation of 3D shrimp surimi gel network (Liu et al., 2022). During heating, myosin undergoes denaturation, expansion, and cross-linking to form an orderly network structure, contributing to shrimp surimi gel quality (Zhang et al., 2023).

Putative filamine-A isoform X3, Arp2/3 complex 34 kDa subunit, and profilin are key component regulating actin network (Francis et al., 2025). Tubulin beta-2 chain is the fundamental structural unit that constitutes microtubules; microtubules, as an important component of the cytoskeleton, can cooperate with actin to form a three-dimensional network (Xu et al., 2023). Meanwhile, compared to the HO group, myosin heavy chain type 1, myosin heavy chain type 2, profilin, and tubulin beta-2 chain were significantly upregulated in the 3% SL group, indicating that SL could effectively inhibit the degradation of these structure-related proteins, thus improving the gel quality of cold-stored *L.* *vannamei*.

#### 3.5.2 Energy metabolism and oxidative stress-related protein

During the cold storage, *L. vannamei* still underwent a series of biochemical reactions, including glycolysis, oxidation, and antioxidant (Huang et a., 2023). In this study, compared with FS group, the content of pyruvate kinase, phosphoglycerate kinase, carnitine O-Palmitoyltransferase, propionyl-CoA carboxylase alpha chain, mitochondrial, and arginine kinase were significantly decreased in HO group.

During cold storage, shrimp muscle exhibited a metabolic shift from aerobic to anaerobic respiration, accompanied by downregulation of key glycolytic enzymes (pyruvate kinase; phosphoglycerate kinase), ultimately suppressing ATP synthesis (Xu et al., 2023). Peng et al. (2019) pointed out that as a rate-limiting enzyme in glycolysis, the downregulation of pyruvate kinase would disrupt the conversion of phosphoenolpyruvate to pyruvate, thus reducing the gel quality of cold-stored *L.* *vannamei*. Meanwhile, suppressed activity of carnitine O-palmitoyltransferase impaired the mitochondrial β-oxidation of long-chain fatty acids. Xu et al. (2023) reported the content of carnitine O-palmitoyltransferase was positive correlation with the hardness of the texture of *L.* *vannamei*. Arginine kinase, as a fast ATP regenerating enzyme in invertebrates, its downregulation would lead to the inability of shrimp cells to maintain energy homeostasis (Guo et al., 2021). The lack of energy accelerated muscle cell death and protein degradation, ultimately deteriorating gel quality of cold-stored *L. vannamei* (Yu et al., 2023).

Meanwhile, compared with HO group, pyruvate kinase, carnitine O-palmitoyltransferase, propionyl-CoA carboxylase alpha chain, mitochondrial, and arginine kinase were significantly increased in 3% SL group. These results indicated that SL could slow down the degradation of myofibrillar protein by protecting energy metabolism, thus maintaining the gel quality of cold-stored *L. vannamei*. The protective effect of SL on metabolic enzymes was likely attributed to its inhibitory activity against the growth of spoilage microorganisms, thereby alleviating the disruption of energy homeostasis in *L.* *vannamei* during cold storage. In addition, SL might enhance the stability of pyruvate kinase through lactylation modification. Support this viewpoint, Liu et al. (2025) reported that SL contributed to maintain the activity of pyruvate kinase in pork during postmortem.

Apart from perturbations in energy metabolism, the structural integrity of shrimp muscle cells was compromised during chilled storage, which in turn triggers dysfunction of the mitochondrial electron transport chain (ETC). This mitochondrial ETC dysfunction induces aberrant electron leakage, ultimately culminating in the excessive production of superoxide anions and subsequent activation of oxidative stress cascades (Zhang et al., 2022). Compared with the FS group, some antioxidant enzymes/proteins in the HO group were significantly down regulated, including glutathione peroxidase, glutathione peroxidase 6, glutathione S-transferase 1, copper/zinc superoxide dismutase isoform 4, hypoxia up-regulated protein. At the same time, compared with the HO group, the SL group significantly upregulated the content of glutathione S-transferase 1, as well as upregulated metallothionein, peroxinnectin, elongation factor 1-alpha. Glutathione peroxidase catalyzes the reduction of hydrogen peroxide and lipid peroxides by glutathione, protecting muscle cells from oxidative damage (Fan et al., 2019). Glutathione S-transferase 1 is involved in clearing endogenous oxidative metabolites such as 4-hydroxynonenal, protecting muscle cell membranes and proteins from lipid peroxidation damage (Du et al., 2024). Copper‑zinc superoxide dismutase (Cu/Zn-SOD) functions as a copper chaperone to maintain superoxide dismutase 1 (SOD1) activity, and its reduced expression would thereby directly compromise the superoxide anion (O₂^−^) scavenging capacity (Guo et al., 2021). Concurrently, hypoxia up-regulated protein modulates oxidative stress responses through HIF-1α stabilization, constituting a critical regulatory axis in cellular redox homeostasis (Fan et al., 2019). Therefore, the protective effects of these antioxidant enzymes/proteins suggested that SL could improve the gel quality of chilled *L.* *vannamei* by alleviating oxidative stress.

While active ATP synthesis ceases post-mortem, residual enzymatic activities—including those of pyruvate kinase and arginine kinase—can influence the rate of ATP depletion and the accumulation of metabolic byproducts (e.g., lactate, H^+^), which in turn affect protease activity and protein denaturation. For instance, rapid ATP depletion leads to a pH decline and disruption of calcium homeostasis, activating calpains and cathepsins. Therefore, the upregulation of these metabolic enzymes in the SL group likely reflects better preservation of cellular integrity during the early post-mortem period, thereby slowing the cascade of proteolytic events that would otherwise degrade myofibrillar proteins.

To verify the alleviation of oxidative stress by SL, we measured TBARS and protein carbonyl content in the FS, HO, and 3% SL groups. As shown in Supplementary 2, the 3% SL group exhibited significantly lower TBARS values (reduced by 41.38% compared to HO, *p* < 0.05) and reduced protein carbonyl content (reduced by 33.47%, p < 0.05), corroborating the upregulation of antioxidant proteins observed in proteomics. These results showed that SL mitigated both lipid and protein oxidation of *L.* *vannamei* during cold storage.

#### 3.5.3 Propholoxidase (proPO) system related proteins

The proPO system is a key component of the innate immune response in *L.* *vannamei* (He et al., 2024). Upon pathogenic infection, this enzymatic system is activated, during which serine proteases cleave inactive proPO into its active form, phenoloxidase (PO). Once produced, PO drives melanin synthesis, generating cytotoxic quinones that disrupt microbial membranes and eliminate spoilage bacteria (He et al., 2024; Hong et al., 2025). In this study, prophenoloxidase-1, prophenoloxidase 2, prophenoloxidase activating factor, and proteins related to the proPO activation system, alpha-2-macroglobulin, putative hemocytin, and JHE-like carboxylesterase 1 were significant decrease in HO vs FS group, while significantly upregulated in SL vs HO group. These results indicate that the proPO defense system of *L.* *vannamei* was severely damaged during cold storage, while SL effectively maintained the proPO system.

It is important to note that these immune-related proteins are not directly involved in gel network formation; rather, their abundance serves as an indicator of the overall health of the muscle tissue prior to processing. By preserving the proPO system, SL reduced microbial pressure and associated stress responses, indirectly safeguarding the structural proteins that are the direct determinants of gel quality.

#### 3.5.4 Immune recognition-related proteins

Pattern recognition proteins (PRPs) act as core components of the innate immune system, functioning as molecular sentinels that detect conserved pathogen-associated molecular patterns (Song et al., 2025). This study observed significant downregulation of PRPs—including C-type lectin 2, C-type lectin-containing domain protein, β-1,3-glucan binding protein, anti-lipopolysaccharide factor 5, and anti-lipopolysaccharide factor VV-R isoform in the HO group compared to the FS group. In contrast, these PRPs were significantly upregulated in the SL group relative to the HO group.

As a member of C-type lectins, a family of key pattern recognition receptors PRRs in crustaceans, CTL2 mediates innate immune responses via its carbohydrate recognition domain (Bi et al., 2020; Peng et al., 2018). As a PRP, β-1,3-glucan-binding protein (β-1,3-GBP) specifically recognizes and binds to β-1,3-glucan moieties in the cell walls of fungi; this binding interaction in turn triggers innate immune responses in *L.* *vannamei* (Abbas et al., 2023; Xiao et al., 2020). Anti-lipopolysaccharide factor, which functions as a lipopolysaccharide (LPS) neutralizer, acts as an immunomodulatory molecule by mitigating LPS toxicity (Qu et al., 2021). Through direct binding to LPS, anti-lipopolysaccharide factor inhibits the interaction between LPS and host immune receptors, thereby suppressing excessive inflammatory responses while preserving immune homeostasis (Wu et al., 2022). Therefore, the reduction in these immune recognition-related proteins in the HO group disrupted the immune homeostasis of *L.* *vannamei*, leading to decreased resistance against spoilage bacteria. In contrast, SL treatment enhanced *L.* *vannamei's* resistance to spoilage bacteria by maintaining the expression levels of these immune recognition-related proteins, thereby improving the gel quality of cold-stored *L.* *vannamei*. During cold storage, the downregulation of immune-related proteins reflects an active immune response to spoilage microbiota, which will compromise muscle integrity through energy reallocation and cellular stress. The upregulation of immune proteins in the SL group suggests that sodium lactate reduces microbial pressure, thereby preserving energy homeostasis and minimizing proteolysis. Thus, the maintenance of immune recognition proteins by SL would help prevent the degradation of myofibrillar proteins caused by microbial spoilage and host immune reactions.

#### 3.5.5 Antimicrobial peptide related protein

Antimicrobial peptides (AMPs) are effective antibacterial agents for *L.* *vannamei* during cold storage. In the present study, six AMPs, penaeidin-3a, penaeidin-3c, crustin I, crustin-like protein, BigPEN, and a putative antimicrobial peptide, were significantly downregulated in the HO group compared to the FS group. Conversely, these six AMPs were significantly upregulated in the SL group relative to the HO group. These findings indicated that the antibacterial capacity of *L.* *vannamei* decreased during cold storage, whereas SL effectively enhanced this antibacterial ability.

The penaeidin family constitutes core AMPs in *L.* *vannamei*, exerting bactericidal effects against both Gram-positive and Gram-negative bacteria via membrane disruption. Notably, our study observed marked reductions in penaeidin-3a and penaeidin-3c levels in HO vs FS group. BigPEN, another member of this family, exhibits potent inhibitory activity against Gram-negative pathogens such as *V. parahaemolyticus* (Xiao et al., 2021). Crustin, a pivotal AMP family in *L.* *vannamei*, inhibits pathogen proliferation by targeting microbial membrane structures, including *Vibrio* spp. and the taura syndrome virus (Barreto et al., 2022). In vitro assays confirm that recombinant crustin A displayed significant antibacterial activity against aquatic pathogens such as *V. parahaemolyticus* (Dai et al., 2023). These AMPs (penaeidins and crustin) are primarily synthesized and stored in hemocytes, with their release triggered by bacterial infection. The observed downregulation in the HO group might result from either extracellular release during cold storage or enzymatic hydrolysis. The upregulation of AMPs in the SL group might be attributed to SL-mediated inhibition of spoilage bacteria growth, thereby reducing AMP consumption (Scheleguedaa, Zalazar, Gliemmo, Campos, 2016). Collectively, these results suggested that SL maintained AMPs activity, thereby improving the gel quality of cold-stored *L.* *vannamei*. The upregulation of antimicrobial peptides in the SL group likely resulted from reduced bacterial growth, which in turn decreased the consumption of these peptides. This preservation of the innate immune barrier further contributes to maintaining muscle integrity during cold storage.

## 4 Conclusion

This study investigated the effect of SL on the gel quality of cold-stored *L. vannamei* and its potential mechanism through physicochemical analysis and 4D-DIA proteomics. Cold storage impaired gel quality (reduced strength/viscosity, higher cooking loss, loose structure, abnormal water migration) due to protein hydrolysis. SL treatment alleviated these defects, with enhancing surimi viscoelasticity, and forming compact gel networks. The results of proteomics indicated that SL preserved structural proteins (e.g., myosin), maintained energy homeostasis (upregulating pyruvate kinase), alleviated oxidative stress (via antioxidants like glutathione S-transferase 1), and preserved immune function (e.g., proPO system, AMPs), thus improving the gel quality of cold-stored *L. vannamei*. Our work provides a comprehensive proteomic landscape of SL's effects on *L.* *vannamei* and highlights the integrated roles of structural preservation, energy metabolism, antioxidant defense, and immune modulation in maintaining the gel quality of *L.* *vannamei* during cold storage. Future research employing functional validation approaches, such as enzyme activity measurements, and targeted inhibitor assays, is necessary to elucidate the direct mechanistic connections between individual proteins and gel properties.

## CRediT authorship contribution statement

**Yantao Yin:** Writing – original draft, Methodology, Data curation. **Huixiang Ye:** Validation, Data curation. **Jia Cai:** Writing – original draft, Methodology. **Caixia Yin:** Writing – original draft, Methodology. **Tengyang Guo:** Writing – original draft, Methodology. **Naiyong Xiao:** Resources, Formal analysis. **Zefu Wang:** Software, Formal analysis. **Shuai Wei:** Resources. **Shucheng Liu:** Writing – review & editing, Supervision, Conceptualization.

## Funding

This work was supported by the Key R&D Program of Guangdong Province (No. 2025B1111140001), Modern Agro-industry Technology Research System of China (No. CARS-48), Doctoral Research Initiation Fee Grant Program, GDOU (060302042315), Innovative experimental project for college students of Guangdong Ocean University (S202510566033).

## Declaration of competing interest

The authors declare that they have no known competing financial interests or personal relationships that could have appeared to influence the work reported in this paper.

## Data Availability

Data will be made available on request.
